# Value of multiparametric MRI in predicting postoperative upgrading of prostate cancer with biopsy Gleason Grade Groups 3 + 3 and 3 + 4

**DOI:** 10.1093/bjro/tzag022

**Published:** 2026-10-19

**Authors:** Jiahui Zhang, Xiaoxiao Zhang, Xin Bai, Li Chen, Erjia Guo, Yongfei Wu, Gumuyang Zhang, Zhengyu Jin, Hao Sun

**Affiliations:** Department of Radiology, State Key Laboratory of Complex Severe and Rare Disease, Peking Union Medical College Hospital, Chinese Academy of Medical Sciences & Peking Union Medical College, Beijing 100730, China; Department of Radiology, State Key Laboratory of Complex Severe and Rare Disease, Peking Union Medical College Hospital, Chinese Academy of Medical Sciences & Peking Union Medical College, Beijing 100730, China; Department of Radiology, State Key Laboratory of Complex Severe and Rare Disease, Peking Union Medical College Hospital, Chinese Academy of Medical Sciences & Peking Union Medical College, Beijing 100730, China; Department of Radiology, State Key Laboratory of Complex Severe and Rare Disease, Peking Union Medical College Hospital, Chinese Academy of Medical Sciences & Peking Union Medical College, Beijing 100730, China; Department of Radiology, State Key Laboratory of Complex Severe and Rare Disease, Peking Union Medical College Hospital, Chinese Academy of Medical Sciences & Peking Union Medical College, Beijing 100730, China; Department of Radiology, State Key Laboratory of Complex Severe and Rare Disease, Peking Union Medical College Hospital, Chinese Academy of Medical Sciences & Peking Union Medical College, Beijing 100730, China; Department of Radiology, State Key Laboratory of Complex Severe and Rare Disease, Peking Union Medical College Hospital, Chinese Academy of Medical Sciences & Peking Union Medical College, Beijing 100730, China; Department of Radiology, State Key Laboratory of Complex Severe and Rare Disease, Peking Union Medical College Hospital, Chinese Academy of Medical Sciences & Peking Union Medical College, Beijing 100730, China; National Center for Quality Control of Radiology, Beijing 100730, China; Department of Radiology, State Key Laboratory of Complex Severe and Rare Disease, Peking Union Medical College Hospital, Chinese Academy of Medical Sciences & Peking Union Medical College, Beijing 100730, China; National Center for Quality Control of Radiology, Beijing 100730, China

**Keywords:** prostatic neoplasms, magnetic resonance imaging, PI-RADS, Gleason Grade Group

## Abstract

**Objectives:**

This study assessed the effectiveness of multiparametric MRI (mpMRI) in predicting postoperative prostate cancer (PCa) upgrading with biopsy Gleason Grade Groups (bGGs) 3 + 3 and 3 + 4.

**Methods:**

We gathered retrospective data from clinical and mpMRI results from 145 patients with PCa. All patients underwent biopsies and then radical prostatectomy (RP). We compared the characteristics of the Gleason Grade Group (GG) upgrading group and nonupgrading group. To assess predictors of GG upgrading after RP, we performed univariable and multivariable logistic regression analyses. Moreover, to determine the prediction efficacy for GG upgrading after RP, we calculated the areas under the curve (AUCs) through a receiver operating characteristic curve analysis.

**Results:**

We observed 83 cases with bGG 3 + 3, and 62 cases with bGG 3 + 4. Of the 145 patients, 81 cases (55.9%) exhibited GG upgrading, while 64 cases (44.1%) did not. The GG upgrading group demonstrated a significantly greater proportion of cases at the base than the nonupgrading group. Moreover, the GG upgrading group exhibited a higher grade of extraprostatic extension than the GG nonupgrading group (both *P *< .05). The clinical model demonstrated an AUC of 0.621, while the mpMRI model showed a higher AUC of 0.707. The composite model incorporating both clinical and mpMRI variables showed enhanced predictive ability (AUC = 0.722) compared with the clinical-only model (*P *= .017).

**Conclusions:**

Compared with standalone clinical assessment, the combined mpMRI-clinical approach enhances prediction accuracy for postoperative GG upgrading, which can decrease undertreatment rates in PCa management.

**Advances in knowledge:**

mpMRI can help predict postoperative PCa upgrading with bGGs 3 + 3 and 3 + 4.

## Introduction

In 112 countries worldwide, prostate cancer (PCa) is the most common cause of male cancer, comprising 15% of all cancers in men.^1^ The Gleason Grade Group (GG) is an important risk stratification and prognostic indicator for PCa.^2^^,^^3^ Patients with GGs 3 + 3 and 3 + 4 can optionally undergo active surveillance (AS). Active surveillance involves conducting regular tests such as prostate-specific antigen (PSA) assessments, digital rectal examination, imaging examinations, and biopsy to closely follow up on patients with PCa. Those with GG 4 + 3 or above are not suited for AS; radical prostatectomy (RP) combined and either radiotherapy or androgen-deprivation therapy are recommended for these patients.^4^^,^^5^

Although the PCa treatment decision traditionally relies on the biopsy GG (bGG),^4^ significant discrepancies (20%-60%) exist between bGG and GG after RP (pGG).^6^ Therefore, precise patient stratification is vital in therapeutic decision-making.^7^^,^^8^ In PCa treatment, avoiding undertreatment is a critical priority, especially for aggressive tumors requiring definitive therapy. Therefore, establishing accurate upgrading-prediction models for bGGs 3 + 3 and 3 + 4 is critical for tumor risk assessment and treatment formulation in patients with PCa.

Magnetic resonance imaging (MRI) is a noninvasive examination with high soft-tissue resolution, making it the preferred imaging modality for PCa diagnosis. Moreover, multiparametric MRI (mpMRI) combines anatomical and functional parameters, thus becoming valuable in PCa detection, biopsy targeting, stratification, and grade estimation.^9^^,^^10^ The Prostate Imaging Reporting and Data System version 2.1 (PI-RADS v2.1) established new research standards to ensure standardization in obtaining and reporting mpMRI data.^11^ Currently, mpMRI is indispensable in PCa diagnosis and management.^12^

For PCa with bGGs 3 + 3 and 3 + 4, lacking reliable preoperative indicators for predicting GG upgrading after RP may lead to insufficient risk assessment and suboptimal patient management. Therefore, this study aimed to determine the utility of mpMRI in predicting post-prostatectomy GG upgrading.

## Methods

### Patients

The institutional review board (IRB) authorized this retrospective study (IRB number I-22PJ1031), which was exempted from patient consent, enabling the collection and analysis of 401 patients with PCa from 2014 to 2022. The patient inclusion criteria were as follows: (1) the patient underwent a complete MRI and laboratory examinations, (2) the PCa diagnosis was established through an ultrasound-guided prostate biopsy and RP after MRI examination. The exclusion criteria were as follows: (1) bGG other than 3 + 3 or 3 + 4 (*n* = 230); (2) the therapy was received before prostate biopsy and RP (*n* = 14); and (3) incomplete clinical or imaging data (*n* = 12). The patient enrollment process is illustrated in Figure 1, which shows a final cohort of 145 participants included in the study.

**Figure 1:**
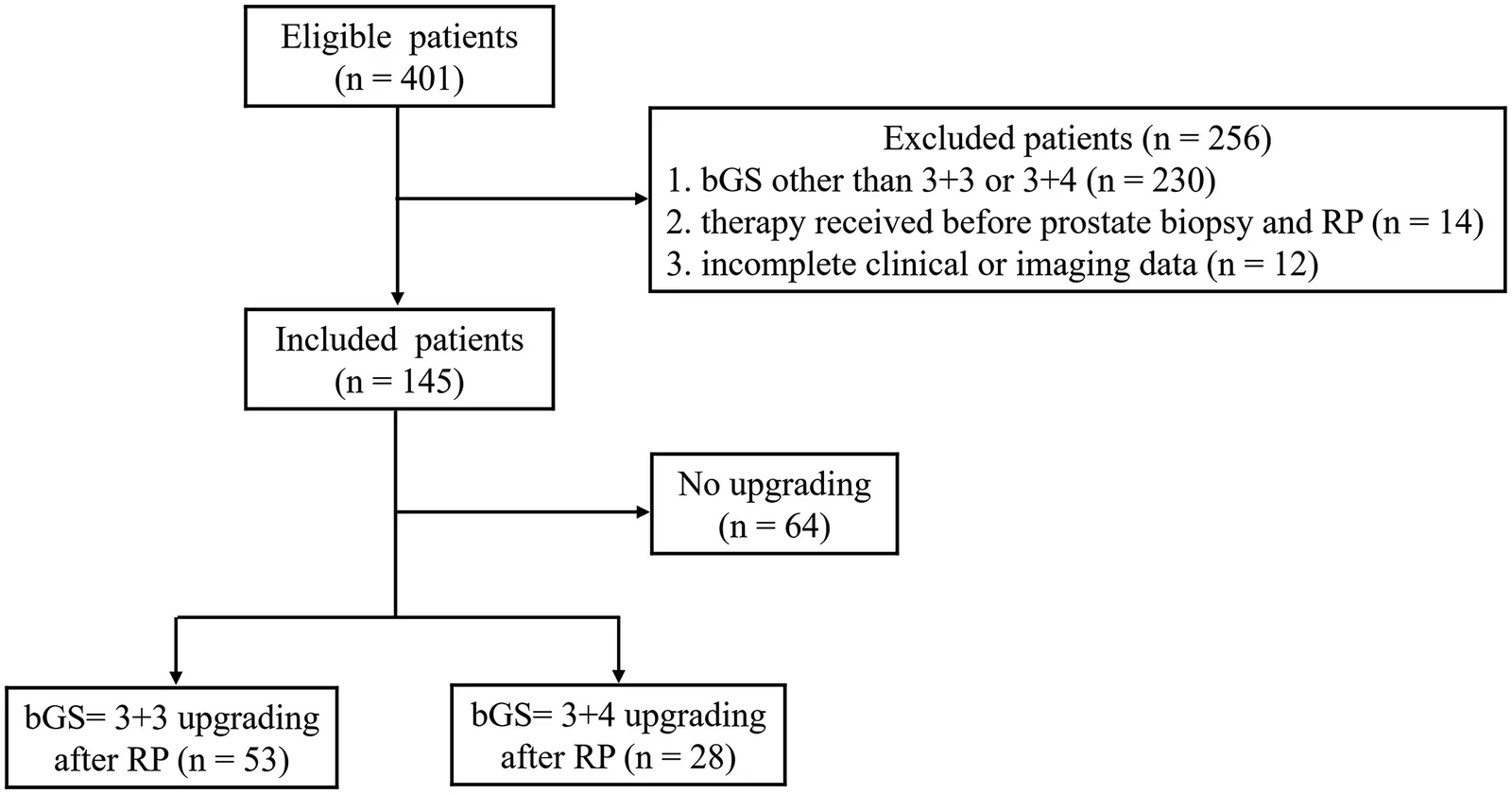
Patient workflow chart. Abbreviation: bGS, biopsy Gleason Grade Group; RP, radical prostatectomy.

### Clinical and pathological data

Clinical and pathological data were collected from patient medical records, including the bGG, pGG, time from biopsy to RP, the number of positive biopsy cores (no. of PBCs), total PSA (tPSA), free PSA (fPSA), and the ratio of free/total PSA (F/T). Prostate-specific antigen values were obtained within 8 weeks before the mpMRI examination. The biopsy protocol consisted of 2 components: (1) an 11-core systematic transperineal approach under ultrasound guidance and (2) cognitive-targeted biopsies based on MRI results. For targeted biopsies, the urologist reviewed pre-procedure MRI images and then sampled the most suspicious nodules under ultrasound guidance.^13^ The prostatectomy specimens were sliced transversely at 3-5-mm intervals from the apex to the base, with tumor margins delineated on each section. Both biopsy and RP specimens were evaluated by a pathologist (a decade of experience) using the standardized Gleason grading system for PCa assessment.^14^ Gleason Grade Groups were defined as follows: 3 + 3, 3 + 4, 4 + 3, 8, 9, and 10.^15^

### MRI protocol

All MRI examinations were administered using a 3.0-T GE750 scanner, following a standardized protocol that incorporated axial T1-weighted image (T1WI), axial/coronal/sagittal T2-weighted image (T2WI), diffusion-weighted imaging (DWI), and dynamic contrast-enhanced sequences. Gadolinium was utilized as the contrast enhancement agent at a flow rate of 3 mL/s and 0.1 mmol/kg. Table 1 lists the MRI image-acquisition parameters. In the study, *b*-values were 0, 50, 100, 150, 200, 500, 800, 1000, 1500, and 2000 s/mm^2^. According to PI-RADS v2.1 criteria, we obtained the ADC maps from *b*-values of 100 and 1000 s/mm^2^, where “high *b*-value” refers to *b*-values of 1500 and 2000 s/mm^2^.

**Table 1:** MRI sequence parameters.

Parameters	T2WI	DWI	DCE
**Sequence**	FRFSE	SE-EPI	3D-GRE
**TR/TE (ms)**	4137/86	4200/90	4.3/1.3
**Flip angle**	110°	90°	12°
**Echo train length**	32	1	N/A
**FOV (mm × mm)**	270 × 270	360 × 360	400 × 400
**Matrix size**	288 × 192	128 × 96	320 × 192
**Slice thickness (mm)**	3.0	3.0	3.0
**Other**		*b* values = 0, 50, 100, 150, 200, 500, 800, 1000, 1500, 2000 s/mm^2^	Temporal resolution <10 s, total scan time of 5 min

Abbreviations: 3D-GRE, 3D-gradient echo; DCE, dynamic contrast-enhanced; DWI, diffusion-weighted imaging; FOV, field of view; FRFSE, fast relaxation fast spin echo; SE-EPI, spin echo echo planar imaging; T2WI, T2-weighted imaging; TE, time echo; TR, repetition time.

### Image analysis

Two blinded genitourinary radiologists (5 and 14 years of experience, respectively) independently interpreted the images. Discrepancies were resolved through consensus review. A third radiologist (4 years of experience) performed MRI-pathology correlation. The MRI parameters were the prostate volume, PSA density (PSAD), PCa diameter, PCa location (peripheral zone/transition zone, base/midgland/apex), extraprostatic extension (EPE) grade and PI-RADS v2.1 scores.

The peripheral and transition zones were differentiated on axial T2WI sequences, with large lesions often spanning both zones. Sagittal T2WI was used to divide the prostate into 3 equal segments along its longitudinal axis: base, midgland, and apex. The grading criteria of EPE based on mpMRI were as follows: (1) grade 0: no obvious capsular contact, or a curvilinear contact length (CCL) < 15 mm; (2) grade 1: CCL ≥ 15 mm, or an irregular or protruding envelope; (3) grade 2: CCL ≥ 15 mm and an irregular or protruding envelope; (4) grade 3: marked capsular breakthrough.^16^ Using mpMRI, the radiologists assessed index lesions across all imaging sequences and ascertained their corresponding PI-RADS v2.1 scores. Lesions with PI-RADS scores of 4 or higher were classified as PCa. Prostrate cancer locations were unavailable and captured as N/A when the PI-RADS score was under 3. When multiple lesions existed, statistical analysis incorporated the lesion demonstrating the highest pGG. Statistical analysis prioritized the largest lesion in cases where pGGs were identical.

### Statistical analysis

All statistical analyses were conducted with R software (version 4.3.1). Continuous variables following a normal distribution were given as mean with SD, and compared using independent samples *t*-tests. For skewed distributions, data were summarized as median with quartiles and analyzed with Mann-Whitney *U* tests. The consistency between the 2 radiologists’ interpretations was measured by kappa statistics (κ), with the agreement levels being categorized as follows: no agreement (κ < 0.20), slight agreement (κ = 0.21-0.40), moderate agreement (κ = 0.41-0.60), strong agreement (κ = 0.61-0.80), and nearly perfect agreement (κ = 0.81-1.00). The chi-squared test was used to assess differences in PCa location, EPE grade, and PI-RADS v2.1 score.

Univariable and multivariable logistic regressions were used to predict GG upgrading after RP. Three models were constructed: a clinical-only model, an mpMRI-only model, and a combined model integrating all variables. The receiver operating characteristic (ROC) curve was employed to assess the area under the curve (AUC). *P*-values <.05 were considered statistically significant.

## Results

### Clinical and pathological data

This study recruited 145 patients with PCa. There were 83 cases with bGG 3 + 3, and 62 cases with bGG 3 + 4. Of the 145 patients, 81 (55.9%) cases experienced GG upgrading, while 64 cases (44.1%) did not. Fifty-three patients (63.9%) with bGG 3 + 3 showed upgrading to pGG ≥ 3 + 4, and 28 patients (45.2%) with bGG 3 + 4 were upgraded to pGG ≥ 4 + 3. The patient age ranged from 46 to 80 years (mean age, 66 years), and the median tPSA value was 7.67 ng/mL. Key symptoms consisted of dysuria, frequent urination, and urinary urgency. A total of 51 (35.2%) patients had EPE. In terms of PI-RADS v2.1 classification, there were 118 patients with category 4 or 5 lesions, 15 patients with category 3 lesions, and 12 patients with category 2 lesions. The inter-reader agreement between the 2 radiologists for mpMRI results was strong, with a κ value of 0.752. Table 2 lists the patients’ characteristics.

**Table 2:** The characteristics of patients.

Patient characteristics	Value
**Age (years), mean ± SD**	66.0 ± 5.8
**Prostate volume (cm^3^), median [IQR]**	36 [16-50]
**tPSA (ng/mL), median [IQR]**	7.67 [5.69-12.28]
**fPSA (ng/mL), median [IQR]**	1.14 [0.82-1.64]
**F/T, mean ± SD**	0.158 ± 0.068
**PSAD (ng/mL/cm^3^), median [IQR]**	0.22 [0.15-0.34]
**Number of positive biopsy cores, median [IQR]**	3 [1-5]
**Time from biopsy to prostatectomy (days), median [IQR]**	32 [23-43]
**PCa diameter (mm), median [IQR]**	10.5 [7.0-14.8]
**PCa location**	
** Peripheral zone**	71
** Transition zone**	59
** Peripheral zone + transition zone**	3
** N/A**	12
**PCa location**	
** Base**	30
** Midgland**	55
** Apex**	48
** N/A**	12
**Extraprostatic extension**	
** Yes**	51
** No**	94
**PI-RADS v2.1 score**	
** 2**	12
** 3**	15
** 4**	78
** 5**	40
**Gleason Grade Group after prostatectomy**	
** 3 + 3**	35
** 3 + 4**	65
** 4 + 3**	27
** 8**	13
** 9-10**	5

When PI-RADS score <3, PCa location was not available and was indicated by N/A. Abbreviation: PI-RADS, Prostate Imaging Reporting and Data System.

### Characteristics of the upgrading and non-upgrading cohorts

Table 3 shows the differences between the GG upgrading group and the GG nonupgrading group. The GG upgrading group exhibited a much larger proportion of cases at the base compared to the nonupgrading group, and the GG upgrading group had a higher grade of EPE than the GG nonupgrading group (both *P *< .05). In the GG upgrading group, 24 (29.6%) patients had the lesions at the base. In contrast, in the GG nonupgrading group, only 6 (9.4%) patients had the lesions at the base. Of the 81 patients with GG upgrading, 19 (23.5%) cases had EPE grade 1, 15 (18.5%) cases had EPE grade 2, 2 (2.4%) cases had EPE grade 3, and 45 (55.6%) showed no EPE. Of the 64 patients with GG nonupgrading, 11 (17.2%) cases had EPE grade 1, 4 (6.2%) cases had EPE grade 2, and 49 (76.6%) showed no EPE.

**Table 3:** Gleason Grade Group upgrading cohort characteristics.

Characteristics	Difference between bGG and pGG		*P-*value
	GG upgrading (*n* = 81)	GG nonupgrading (*n* = 64)	
**Age (years), mean ± SD)**	66.3 ± 4.7	65.5 ± 7.0	.436
**Prostate volume (cm^3^), median [IQR]**	33 [26-52]	39 [29-49]	.447
**tPSA (ng/mL), median [IQR]**	7.38 [5.32-12.50]	8.09 [6.26-11.00]	.361
**fPSA (ng/mL), median [IQR]**	1.14 [0.82-1.76]	1.16 [0.83-1.58]	.817
**F/T, mean ± SD**	0.160 ± 0.074	0.155 ± 0.060	.656
**PSAD (ng/mL/cm^3^), median [IQR]**	0.24 [0.15-0.35]	0.21 [0.14-0.32]	.226
**Number of positive biopsy cores, median [IQR]**	3 [2-5]	2 [1-5]	.306
**Time from biopsy to prostatectomy (days), median [IQR]**	33 [22-47]	32 [24-40]	.800
**PCa diameter (mm), median [IQR]**	11.1 [7.0-15.5]	10.0 [6.8-14.6]	.117
**PCa location**			.249
** Peripheral zone**	45 (55.6%)	26 (40.6%)	
** Transition zone**	29 (35.8%)	30 (46.9%)	
** Peripheral zone + transition zone**	2 (2.4%)	1 (1.6%)	
** N/A**	5 (6.2%)	7 (10.9%)	
**PCa location**			.015
** Base**	24 (29.6%)	6 (9.4%)	
** Midgland**	27 (33.3%)	28 (43.8%)	
** Apex**	25 (30.9%)	23 (35.9%)	
** N/A**	5 (6.2%)	7 (10.9%)	
**Extraprostatic extension grade**			.032
** 0**	45 (55.6%)	49 (76.6%)	
** 1**	19 (23.5%)	11 (17.2%)	
** 2**	15 (18.5%)	4 (6.2%)	
** 3**	2 (2.4%)	0 (0%)	
**PI-RADS v2.1 score**			.728
** 2**	5 (6.0%)	7 (11.0%)	
** 3**	8 (10.0%)	7 (11.0%)	
** 4**	44 (54.0%)	34 (53.0%)	
** 5**	24 (30.0%)	16 (25.0%)	

When PI-RADS score <3, PCa location was not available and was indicated by N/A. Abbreviations: bGG, biopsy Gleason Grade Group; F/T, ratio of free/total PSA; fPSA, free PSA; OR, odds ratio; PCa, prostate cancer; pGG, Gleason Grade Group after radical prostatectomy; PI-RADS, Prostate Imaging Reporting and Data System; PSAD, PSA density; tPSA, total PSA.

Figure 2 illustrates a case of GG upgrading in a 62-year-old male patient, with GG increasing from 3 + 3 at biopsy to 4 + 3 after RP. Figure 3 illustrates a case of GG upgrading in a 66-year-old male patient, with GG increasing from 3 + 4 at biopsy to 4 + 5 after RP.

**Figure 2:**
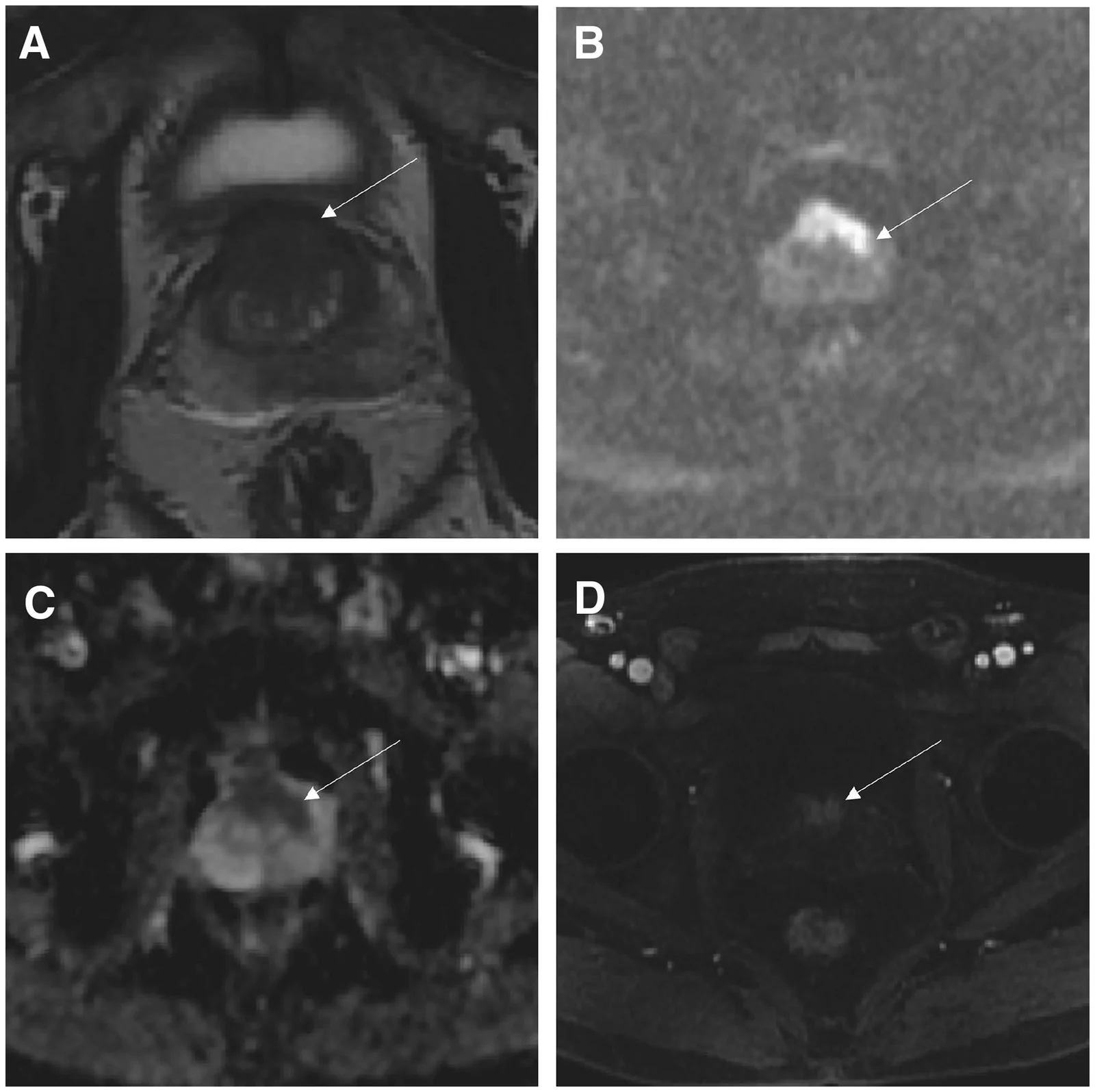
A 62-year-old man experienced GG upgrading from 3 + 3 at biopsy to 4 + 3 after radical prostatectomy (RP). (A) The axial T2-weighted imaging (T2WI) demonstrated a focal hypointense lesion (approximately 19.4 mm) at the base in the transitional zone, assigned a T2WI score of 5, along with grade 2 extraprostatic extension. (B, C) Diffusion-weighted imaging (DWI) demonstrated marked hyperintensity in the lesion with corresponding hypointensity on ADC map, scoring 5 points. (D) Dynamic contrast-enhanced (DCE) score was positive. The PI-RADS, Prostate Imaging Reporting and Data System version 2.1 (PI-RADS v2.1) score was 5 points.

**Figure 3:**
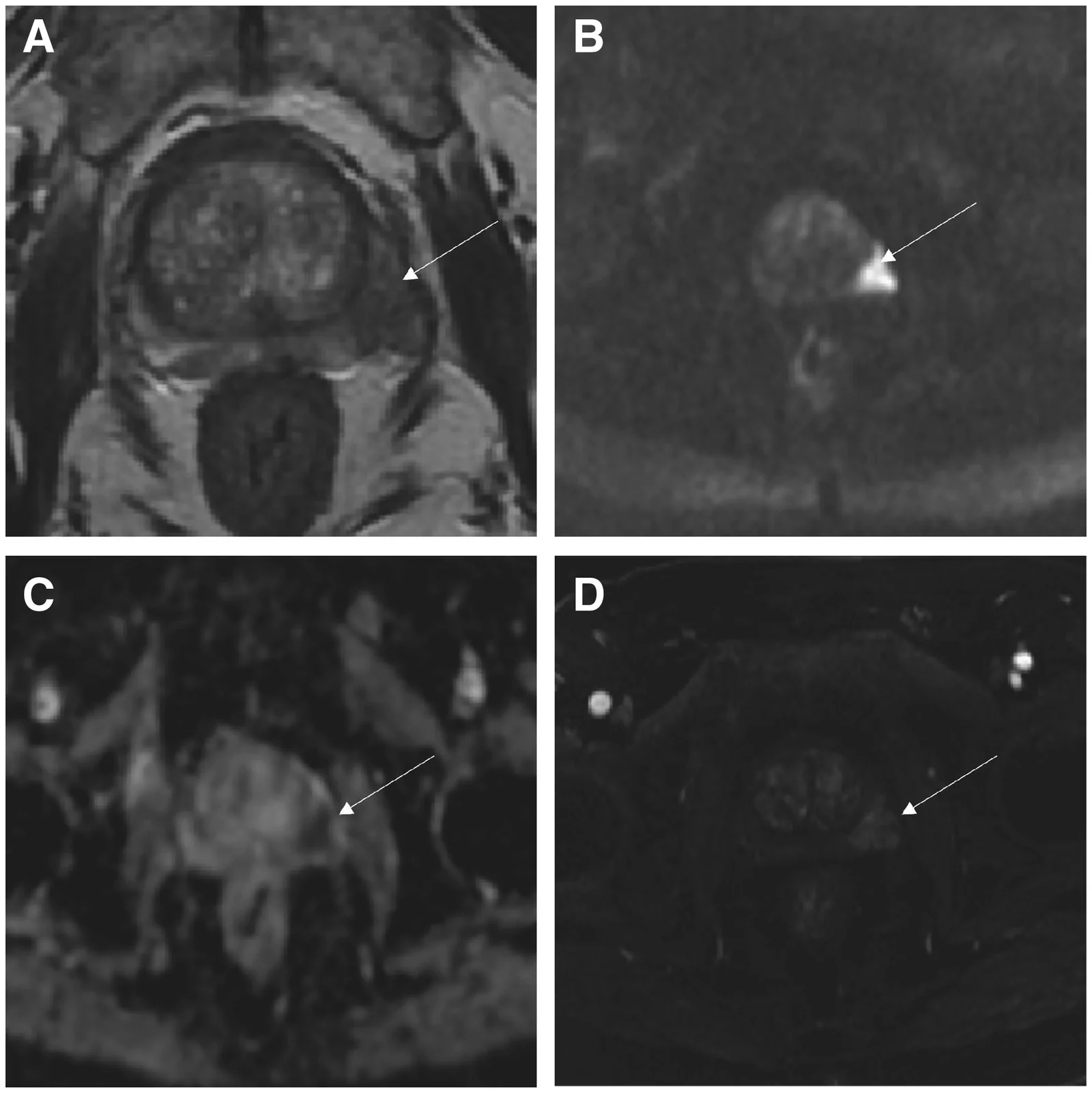
A 66-year-old man experienced GG upgrading from 3 + 4 at biopsy to 4 + 5 after radical prostatectomy (RP). (A) The axial T2-weighted imaging (T2WI) demonstrated a focal hypointense lesion (approximately 15.8 mm) at the base in the peripheral zone, assigned a T2WI score of 5, along with grade 2 extraprostatic extension. (B, C) Diffusion-weighted imaging (DWI) demonstrated marked hyperintensity in the lesion with corresponding hypointensity on ADC map, scoring 5 points. (D) Dynamic contrast-enhanced (DCE) score was positive. The PI-RADS, Prostate Imaging Reporting and Data System version 2.1 (PI-RADS v2.1) score was 5 points.

### Logistic regression analyses and diagnostic efficacies

Factors predictive of GG upgrading after RP were assessed using both univariable and multivariable logistic regression analyses, with results shown in Table 4. To assess the predictive performances of clinical and mpMRI variables for GG upgrading after RP, we constructed ROC curves of clinical factors, mpMRI findings, and the combined diagnosis model incorporating all the clinical and mpMRI variables (Figure 4). The clinical factors included age, time from biopsy to RP, no. of PBCs, tPSA, fPSA, F/T, prostate volume, and PSAD. The mpMRI findings included PCa diameter, PCa location, EPE grade, and PI-RADS v2.1. The clinical model demonstrated an AUC of 0.621, while the mpMRI model showed a higher AUC of 0.707. The composite model incorporating both clinical and mpMRI variables demonstrated enhanced predictive ability (AUC = 0.722) compared to the clinical-only model (*P *= .017), with results shown in Table 5.

**Figure 4:**
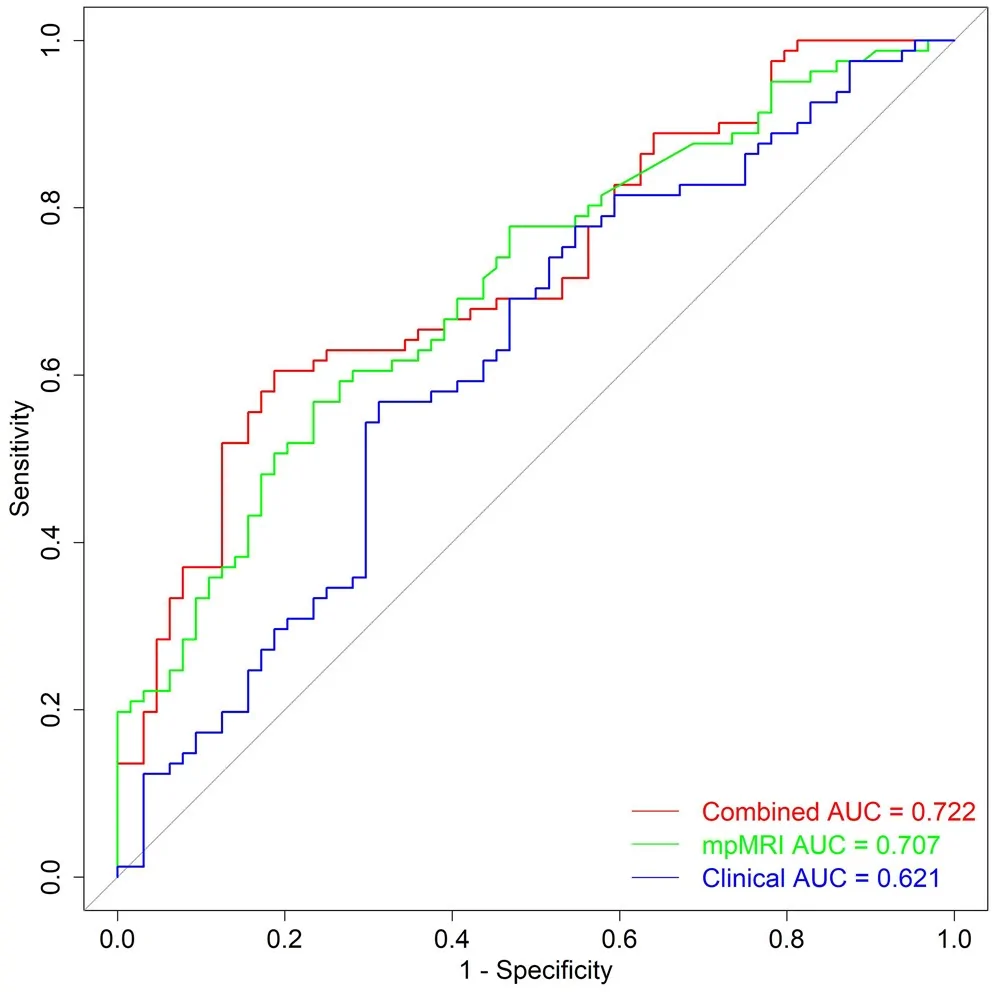
The combined diagnostic model achieved superior predictive accuracy for Grade Group (GG) upgrading after radical prostatectomy (RP) (area under the curve [AUC] = 0.722), compared with the multiparametric MRI (mpMRI) model (AUC = 0.707) and clinical model alone (AUC = 0.621).

**Table 4:** Logistic analysis results affecting Gleason Grade Group upgrading after prostatectomy.

Characteristics	Univariable analysis			Multivariable analysis								
				Clinical model			mpMRI model			Combined model		
	OR	OR 95%CI	*P-*value	OR	OR 95%CI	*P-*value	OR	OR 95%CI	*P-*value	OR	OR 95%CI	*P-*value
**Age (years)**	1.024	0.968 to 1.085	.413	1.038	0.975 to 1.107	.248				1.059	0.989 to 1.138	.107
**Number of positive biopsy cores**	1.021	0.890 to 1.173	.771	0.956	0.818 to 1.115	.563				0.952	0.799 to 1.131	.574
**Time from biopsy to prostatectomy (days)**	1.002	0.988-1.017	.799	1.002	0.987-1.018	.768				1.003	0.987-1.019	.729
**tPSA (ng/mL)**	1.027	0.969-1.094	.376	1.115	0.947-1.310	.193				1.047	0.860-1.273	.643
**fPSA (ng/mL)**	0.993	0.755-1.318	.956	0.601	0.276-1.205	.158				0.530	0.224-1.250	.133
**F/T**	0.318	0.003-31.76	.623	89.02	0.018-6.0E5	.305				206.6	0.012-4.0E5	.284
**Prostate volume (cm^3^)**	0.993	0.977-1.010	.435	0.990	0.967-1.020	.477				0.998	0.971-1.030	.886
**PSAD (ng/mL/cm^3^)**	3.184	0.921-24.16	.233	2.129	0.879-124.2	.680				4.149	0.827-353.2	.646
**PCa diameter (mm)**	1.039	0.990-1.095	.128				1.013	0.929-1.109	.778	1.022	0.932-1.128	.518
**PCa location (base vs midgland/apex)**	4.070	1.637-11.66	.004				4.199	1.578-12.84	.007	4.745	1.638-15.91	.007
**PCa location (peripheral zone/transition zone)**	1.617	0.989-2.696	.059				1.727	0.938-3.254	.083	1.845	0.956-3.646	.071
**Extraprostatic extension grade**	2.061	1.284-3.501	.004				2.233	1.240-4.246	.010	2.405	1.277-4.777	.009
**PI-RADS v2.1 score (ranked category)**	1.239	0.839-1.845	.282				0.605	0.287-1.222	.171	0.549	0.249-1.160	.124

Abbreviations: F/T, ratio of free/total PSA; fPSA, free PSA; OR, odds ratio; PCa, prostate cancer; PI-RADS, Prostate Imaging Reporting and Data System; PSAD, PSA density; tPSA, total PSA.

**Table 5:** The diagnostic efficacies of 3 models for Gleason Grade Group upgrading after prostatectomy.

Group	AUC (95% CI)	Sensitivity	Specificity	Positive predictive value	Negative predictive value
**Combined model (clinical + mpMRI)**	0.722 (0.640-0.805)	0.605	0.188	0.600	0.400
**mpMRI model**	0.707 (0.623-0.790)	0.568	0.312	0.561	0.439
**Clinical model**	0.621 (0.528-0.714)	0.281	0.605	0.566	0.434

Abbreviations: AUC, the area under the curve; mpMRI, multiparametric MRI.

## Discussion

The bGG is an important factor to assess the biological behavior of PCa and one of the most important indicators for selecting treatment options before RP.^17^ Nevertheless, it should be noted that bGG is limited by sampling constraints, whereas prostatectomy specimens allow for more comprehensive and precise pathological grading. Although needle biopsy technology is constantly improving, inconsistencies between bGG and pGG are frequently reported.

Epstein et al reported that 36.3% of cases were upgraded from bGG ≤ 6 to a higher grade at RP.^18^ Yang et al enrolled approximately 10 000 patients with PCa to investigate the predictors for PCa of GG 7 (3 + 4) upgrading and upstaging; the results demonstrated that approximately 33% of cases with bGG 7 (3 + 4) were upgraded to a higher grade at RP.^19^ In this study, a total of 53 patients (53/83, 63.9%) were upgraded from bGG 3 + 3 to pGG ≥ 3 + 4, and 28 patients (28/62, 45.2%) were upgraded from bGG 3 + 4 to pGG ≥ 4 + 3, which are relatively higher rates than in other studies.^18^^,^^19^ All patients in our study underwent ultrasound-guided systematic and cognitive-targeted biopsies, not MRI-targeted biopsies. There are some cases where urologists and radiologists may have inconsistent interpretations of the images. The urologist decided to biopsy suspicious nodules, potentially introducing bias and contributing to the high upgrading rates in this study. Differences in sample size and race might also account for this divergence. Zhang et al reported a 1.8% incidence rate of GG downgrading from bGS ≥ 7 to pGS ≤ 6,^20^ which indicated that targeted biopsy may reduce GG upgrading, although not commonly.

Evidence from various studies indicates that the integration of preoperative clinical and MRI variables can effectively predict GG upgrading after RP.^6^^,^^21^ Notably, several studies have indicated that the PI-RADS score ≥4 is an independent predictor for GG upgrading.^22–24^ For instance, Wei et al demonstrated that higher preoperative PSA levels independently predicted GG upgrading after RP, with a direct correlation between PSA values and the extent of GG increase compared with the biopsy.^25^ According to Chung et al, smaller prostate volumes were linked to an elevated risk of GG upgrading after RP.^26^ Zhang et al reported that a longer delay between biopsy and RP was associated with an increased risk of GG upgrading.^15^ The present results diverge from those reported in previous investigations. No significant correlations were found among PI-RADS v2.1 score, PSA, prostate volume, time from biopsy to RP, and GG upgrading after RP. Whether these differences in results are due to biological effects or different sample sizes remains unclear.

Our study also revealed that the tumors at the base had a higher likelihood of GG upgrading after RP. We propose that this may be due to the technical difficulty in sampling the tumors at the base during biopsy, compared to the tumors at the midgland and apex, which are more readily accessible. The GG upgrading group was more likely to have EPE and had a higher grade of EPE than the GG nonupgrading group. This result confirms that the GG upgrading group has a worse postoperative prognosis, so these patients are not suited for AS, in contrast to the GG nonupgrading group. Wan et al demonstrated that mpMRI combined with clinical variables achieved superior predictive accuracy for GG upgrading in the patients with a bGG of 6 compared with clinical factors alone.^6^ The predictive performances of clinical and mpMRI variables for GG upgrading after RP were moderate in this study. When clinical and mpMRI variables were integrated, diagnostic efficiency was markedly optimized, which was consistent with the results.^6^ It suggested that MRI can provide important information on PCa prognosis, meaning that it should be incorporated into clinical decision-making.

Active surveillance is now established as the standard approach for treating low-risk PCa to avoid excessive treatment, and most patients with GG 6 are candidates for this management strategy.^27^^,^^28^ When the patient group is accurately selected, treatment by AS is successful, while avoiding excessive treatment and reducing treatment-related complications. For AS to be successful, a comprehensive monitoring plan must be formulated, and continuous cooperation from the patients must be obtained.^29^ While some patients with GG 3 + 4 may qualify for AS, others require definitive treatment. These tumors exhibit prognostic characteristics that are intermediate between GG 3 + 3 and GG 4 + 3.^30^ A previous study has shown that the recurrence rate of PCa with pGG 7 varies considerably, depending on the primary Gleason pattern, with a 5-year biochemical risk-free survival rate of 88% for PCa with GG 7 (3 + 4), but only 63% for GG 7 (4 + 3).^9^

There are several limitations in this study. First, as it was a single-institution retrospective analysis, the possibility of selection bias cannot be ruled out. And the generalizability and robustness of the model remain uncertain. Second, although all patients underwent ultrasound-guided systematic and cognitive-targeted biopsies, there are some cases where urologists and radiologists may have inconsistent interpretations of the images. Third, compared with PI-RADS v2.1 guidelines, T2WI and DWI parameters have a larger field of view. Last, although axial histological sections were aligned with axial mpMRI, morphological discrepancies between histology and imaging persisted, reflecting a common limitation in PCa radiology-pathology studies.

## Conclusions

In this study, it was found that mpMRI can help predict postoperative PCa upgrading with bGGs 3 + 3 and 3 + 4. Compared with standalone clinical assessment, the combined mpMRI-clinical approach can improve the prediction accuracy for postoperative GG upgrading, which can decrease undertreatment rates in PCa management.
